# Automatic Diabetic Foot Ulcer Recognition Using Multi-Level Thermographic Image Data

**DOI:** 10.3390/diagnostics13162637

**Published:** 2023-08-10

**Authors:** Ikramullah Khosa, Awais Raza, Mohd Anjum, Waseem Ahmad, Sana Shahab

**Affiliations:** 1 Department of Electrical and Computer Engineering, COMSATS University Islamabad, Lahore Campus, Lahore 54000, Pakistan; 2 Department of Computer Engineering, Aligarh Muslim University, Aligarh 202002, India; 3 Department of Computer Science and Engineering, Meerut Institute of Engineering and Technology, Meerut 250005, India; 4 Department of Business Administration, College of Business Administration, Princess Nourah Bint Abdulrahman University, P.O. Box 84428, Riyadh 11671, Saudi Arabia

**Keywords:** diabetes mellitus, diabetic foot ulcer, thermograms, deep learning, machine learning

## Abstract

Lower extremity diabetic foot ulcers (DFUs) are a severe consequence of diabetes mellitus (DM). It has been estimated that people with diabetes have a 15% to 25% lifetime risk of acquiring DFUs which leads to the risk of lower limb amputations up to 85% due to poor diagnosis and treatment. Diabetic foot develops planter ulcers where thermography is used to detect the changes in the planter temperature. In this study, publicly available thermographic image data including both control group and diabetic group patients are used. Thermograms at image level as well as patch level are utilized for DFU detection. For DFU recognition, several machine-learning-based classification approaches are employed with hand-crafted features. Moreover, a couple of convolutional neural network models including ResNet50 and DenseNet121 are evaluated for DFU recognition. Finally, a CNN-based custom-developed model is proposed for the recognition task. The results are produced using image-level data, patch-level data, and image–patch combination data. The proposed CNN-based model outperformed the utilized models as well as the state-of-the-art models in terms of the AUC and accuracy. Moreover, the recognition accuracy for both the machine-learning and deep-learning approaches was higher for the image-level thermogram data in comparison to the patch-level or combination of image–patch thermograms.

## 1. Introduction

**Figure 1:**
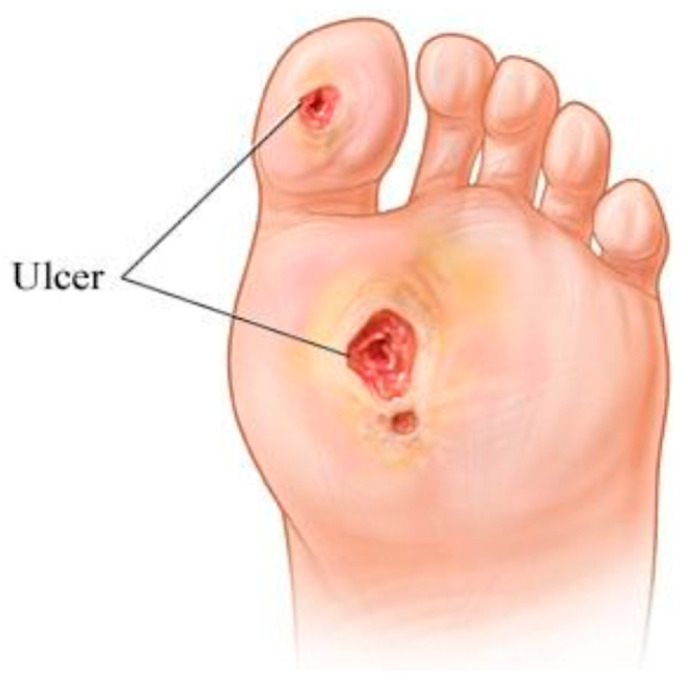
An illustration of foot ulcers.

Insulin insufficiency in the body causes diabetes mellitus (DM), which results in high blood glucose (hyperglycemia) for an extended period. Uncontrolled diabetes for a long period of time can lead to complications such as nephropathy, retinopathy, Charcot foot development, amputation, or even death [1]. Uncontrolled DM damages the nerves; if the nerves in the legs or feet are damaged, it causes a lack of feeling called sensory diabetic neuropathy. When a patient does not feel a sore or cut in his foot due to neuropathy, that cut causes infection and worsens the foot condition. The other situation is the low flow of blood. Peripheral vascular disease causes low blood flow in the arms and legs. If the cut is not healing due to low blood flow, there is a risk of developing ulcers. DFU (diabetic foot ulcer) is most common in diabetic patients; more than 15% of patients face this problem [2]. An illustration of DFU is shown in Figure 1.

Diabetic foot issues are expensive and have a negative impact on one’s quality of life. This may be prevented or considerably delayed in many situations by undertaking a risk assessment and inspection of diabetes patients’ foot health at an early stage. For that purpose, temperature may have an impact. Diabetics’ plantar foot temperature may fluctuate due to neuropathy, ischemia, or infection. Temperature differences of more than 2.2 °C (4 °F) between the right and left foot are considered abnormal, where the normal difference is typically less than 1°C [3,4,5]. With the use of a thermal-imaging camera, problems may be identified early, saving time and money in the long run. Infrared thermography may be utilized to produce a clear image of the thermal energy released by the site being monitored in real time if the temperature is above absolute zero [6,7,8]. Thermography is a non-invasive, non-contact, cost-effective, rapid, and painless means of screening the patient’s skin temperature. This imaging technique can detect temperature changes on human skin.

To detect the ulcer, the thermogram needs to be assessed by a professional expert. The availability of such experts is a challenge, particularly in remote areas. Therefore, several research studies have targeted the provision of an automatic DFU recognition system. Few among them utilized foot thermograms [9,10] while many considered visible-band (RGB camera) images [11,12,13,14,15,16,17,18]. In this study, the thermogram images of diabetic foot are considered for DFU detection. For the recognition of DFU, the experiments are carried out at three levels of thermogram data: image-level, patch-level, and a combination of image–patch thermogram data. To detect the DFU foot, pre-trained deep-learning models are employed using transfer learning. Moreover, a problem-oriented, custom, CNN-based, computationally light model is developed to compare with the state-of-the-art results. For a comprehensive and comparative analysis, classical feature-based recognition using machine-learning techniques is also carried out. The details of the rest of the paper are as follows: Section 2 presents the background work and related studies; Section 3 includes the dataset and augmentation details. The methodology is discussed in Section 4. The results and discussions are presented in Section 5. The conclusion is added in Section 6.

## 2. Background

Machine-learning and deep-learning techniques have gained a lot of interest in recent years for diabetic foot ulcer monitoring and diagnosis in patients with neuropathic diabetes. Much research has been carried out recently for DFU recognition and classification [12,13,15,16,18,19]. However, those studies considered the visible-band images of diabetic feet. Most of these studies employed deep-learning approaches for DFU diagnosis. In contrast, fewer studies judged the thermographic image data. Since this study is focused on the thermographic image data, the literature related to DFU classification in thermograms is discussed.

In the study [9], the researchers compared different machine-learning and deep-learning models. Automatic segmentation and ROI with feature extraction were represented by a fuzzy entropy set with a histogram-based segmentation method for optimization. After augmentation, the data were trained and tested. The SVM produced the lowest AUC (area under the curve), sensitivity, and accuracy. The proposed network model DFTNet and the common ANN had the best performances, using AUC values of 0.8533 and 0.8333%, respectively. In a study by [20], the quantity of pixels with a temperature exceeding 2.2 °C was measured to compute the region of interest. The produced segmentation masks were 99.25% accurate in detecting the absence of a foot sole, 98.83% accurate in constructing a bounding box, and 94.95% accurate in detecting the presence of a foot sole. In another study [14], the authors collected the IRT (infrared thermograph) images and made a dataset with 39 ischemic DFU patients, of which 14 had active ischemic wounds and the remainder had healing wounds. The image ROI abstract was classified by ANN, kNN (k-nearest neighbor), and SVM (support vector machines), while the image was decomposed using DST (discrete wavelength transform) and HOS (higher-order spectra). The best achieved accuracy reported was 98.39% using the SVM classifier. Researchers in study [21] took thermograms in controlled environments with a homogeneous background and used k-means clustering, and a further approach on every foot for foot segmentation. The identification of ulceration on the image was different pixel to pixel and a thresholding technique was used. They successfully differentiated between ulcer and non-ulcer wounds with the help of a classifier and segmented based on a machine-learning model with 91.8% sensitivity, 98.4% specificity, and 91.1% accuracy. In another study [22], the authors used a controlled environment with a room temperature of 20 °C for thermogram images and foot segmentation. A temperature threshold matrix was created, as well as an additional method established to label each foot. A pattern spectrum with thresholding techniques was used to identify the ulceration in the image. They claimed results between the risk and non-risk zones with the help of a classifier and performed segmentation based on a machine-learning model with a sensitivity of 97.33% and specificity of 91.33%. In another study [23], analysis of DFU thermograms with machine learning was performed where the researcher used controlled environments at a room temperature of 20 °C and humidity of 55% for thermogram and foot segmentation. The health-care expert identified the ulceration in the image. The SVM classifier and wavelet characteristics vector were used. They produced results between DF and non-DF with the help of a classifier with an accuracy of 89.39%, sensitivity of 81.81%, and specificity of 96.97%. The detection of DFU thermograms with machine learning has been presented in [24], in an uncontrolled environment with respect to room temperature, illumination, and close-ups for the image of the thermogram. With the temperature filter, the average temperature and threshold were used for diagnostic purposes with the help of a machine-learning-based classifier. They claimed results between DF and non-DF based on a machine-learning model with a non-risk class sensitivity of 91.32% and specificity of 91.84% and ulcer class sensitivity of 90.29%, accuracy of 90.28, and specificity of 90.28%. The authors in study [10] used infrared imaging to detect abnormalities in foot segmentation and registration. They concluded that the ACWE (active contour without edges) method produced quite good results. Automatic pre-symptomatic ulcer detection was performed to determine the clinically relevant difference in temperature between the feet, which was 2.2 °C. The researchers in [7] used infrared imaging and for image decomposition, they examined the ROI of complete feet and mean temperatures. In individuals with localized difficulties, the ipsilateral and contralateral foot and mean temperatures are the same. When compared to a similar area in the contralateral foot and the mean of the entire ipsilateral foot, the ROI temperature was greater than 2 °C. The average temperature difference between both ipsilateral and contralateral feet was greater than 3 °C in patients with widespread problems. In study [25], infrared imaging and clinical foot assessments were presented. For image decomposition, they examined the ROC curve. With a 76% sensitivity and 40% specificity, the contralateral locations had a difference of 2.2 °C between each other, which showed the best cut-off value for diagnosing diabetic foot. The variation of 3.5 °C between the mean temperature of the right and left foot was shown to be the best cut-off value for determining the urgency for treatment, with an 89% sensitivity. In this study [26], the authors employed infrared imaging to detect anomalies, followed by grayscale characterization and temperature pattern foot segmentation. Then, to pattern the spectrum, mathematical morphology was used as well as a multi-layer perceptron with k-fold validation. The subjects had a butterfly pattern, and the pattern spectrum was like that of ovals and rounds. Quadrant 4 had the greatest mean percentage of pixels for the control group, at 88.05%. Due to the different patterns, the pattern spectrum was abnormal. In quadrant 3, the mean proportion of pixels for the diabetic group was 28.87%, while the authors achieved an average classification rate of 94.33 percent. In this paper [27], they examined a database of dynamic IRT plantar diagnostic images with 39 current diabetic foot ulcer patients. The mean temperature of the region of interest, which corresponds to the important change places of diabetic foot ulcer, was assessed and the images were examined by assessing the mean temperature of the region of interest, which relates to some of the important change places of diabetic foot ulcer. The statistics found no evidence of a significant difference between the thermal asymmetry values and thermal recovery differences in any region of interest, except the one at the medial forefoot. The regions of interest were assessed on both feet, with the value of the thermal asymmetry factored into each one. A decision support system was constructed using the database and analytical results to classify the data and examine the accurate identification of the DFU using machine-learning methods such as ANN, kNN, and SVM. The best overall results were achieved with a kNN of 5 neighbors.

## 3. Data and Augmentation

**Figure 2:**
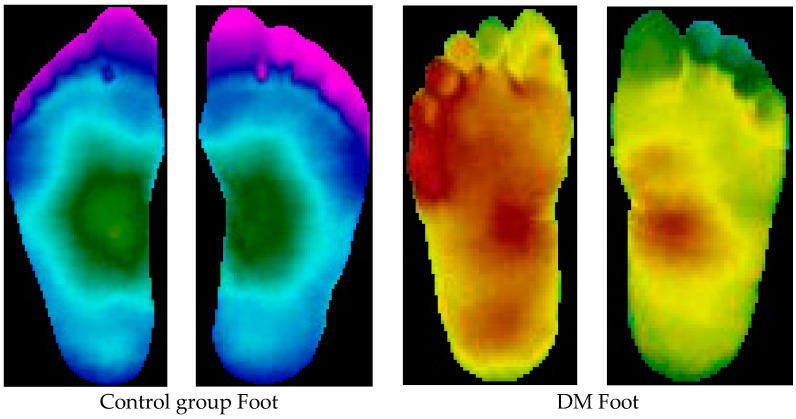
Sample images of healthy foot and DM foot.

A public dataset of thermograms is used in this study [3]. These data comprise 334 plantar thermograms obtained from 122 individuals diagnosed with diabetes mellitus (DM) and 45 individuals not diagnosed with diabetes (control group). In the DM group, there were 16 female and 29 male subjects aged between 20 and 35 years. Moreover, there were 89 females and 33 males with their age ranging from 45–65 years in the control group. The subjects were recruited as volunteers from the city of Puebla, Mexico, and thermogram acquisition was carried out over a period of three years (2012–2014). A sample pair of feet from each group is shown in Figure 2.

**Figure 3:**
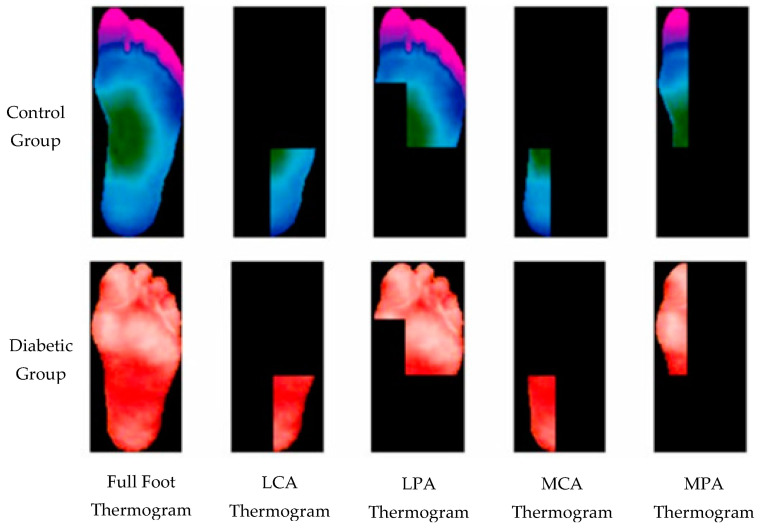
A sample of thermograms from diabetic and control group and corresponding patch images (plantar angiosomes). MPA: medial plantar artery, LPA: lateral plantar artery, MCA: medial calcaneal artery, LCA: lateral calcaneal artery.

Each thermogram includes four more images representing plantar angiosomes. Those are considered as patches in this study. A sample of full images and corresponding patch images (plantar angiosomes) from both categories are shown in Figure 3. For infrared (IR) image acquisition, the subject was laid on a bed with an IR camera at a distance of one meter from the feet [3]. To avoid sensing the temperature from the rest of the body, an IR obstructive material was placed. Two IR camera FLIR E60 and FLIR E6 were used at a room temperature of 20 ± 1 C [3]. Since the database includes the segmented foot and patch RGB images, those are used as they are without any pre-processing. However, data augmentation is utilized to increase the dataset size as well as to balance the classes. Augmentation is carried out by rotating the images at 90°, 180°, and 270°, as well as by horizontal flip, vertical flip, and both horizontal and vertical flip simultaneously. Image-level augmentation is performed by making 500 samples for each class and patch-level augmentation is performed to prepare 1500 samples per class. The class-wise detail of the thermograms is summarized in Table 1.

## 4. Methodology

Computer-aided diagnostic techniques assist medical practitioners in being able to diagnose with a higher confidence. Machine-learning and deep-learning techniques have been of interest for utilization as a decision support system.

### 4.1. Machine-Learning Approaches

Traditional machine-learning techniques have been considered for the classification of thermograms in the literature. Based on their proven performance in the literature, including medical diagnosis, several of them were considered for this study for comprehensive comparative analysis including SVM, random forest (RF), multi-layer perceptron (MLP), naive Bayes, kNN, XGBoost, AdaBoost, and bagging.

**Support Vector Machines:** Support vector machine may be utilized both for regression and classification problems [28]. For classification, it is a classifier with a goal to locate a hyperplane separating the two classes with a large margin.
**Random Forest:** It is a classifier with several decision trees on various subsets of the provided dataset that takes the average to enhance the predicted accuracy of that dataset [29]. To anticipate the ultimate output, the random forest collects guesses from all of its trees and combines them into a single prediction. Overfitting can be avoided by having a larger number of trees to choose from when making a model.
**kNN:** k-nearest neighbor algorithm assigns the class to the test sample based on the nearest neighbors with the largest majority [30]. Being nearest depends on the distance metric which is normally the Euclidean distance or absolute distance.
**Naive Bayes:** To categorize the data, a naive Bayes classifier applies concepts from probability theory [31]. The theorem developed by Bayes is utilized by the naive Bayes classification algorithms. The most important takeaway from Bayes’ theorem is that the probability of an event can be recalculated whenever new evidence is added to the mix.
**XGBoost:** Extreme gradient boosting is built on supervised machine learning, decision trees, ensemble learning, and gradient boosting [32].
**AdaBoost:** As part of an ensemble method in machine learning, adaptive boosting is a technique known as AdaBoost [33]. AdaBoost’s most frequent algorithm is a decision tree with only one split, known as a decision tree with only one level. Decision stumps are another name for these trees. This algorithm creates a model and equally weighs all the input data points in that model.
**Bagging:** An ensemble meta-estimator, bagging classifiers fit base classifiers on random subsets of the original dataset and then aggregate their individual predictions (either by voting or average) to generate a final forecast [34].

### 4.2. Feature Extraction

The machine-learning classifiers discussed above require feature extraction. There are many features in the literature that have been used for computer vision and pattern recognition tasks; a few of the popular ones among them are used in this study including local binary pattern, gray level cooccurrence matrix, histogram of oriented gradients, and Gabor features to be used with machine-learning classifiers.

**Local Binary Patterns (LBP):** Each pixel in a picture is labelled using the local binary patterns operator by thresholding a 3x3 neighborhood surrounding each pixel with the center value [35]. These classes are used to label pixels. Each result is assigned a binary value, which is either a 1 or a 0, depending on whether the surrounding pixels are equal or greater than the center pixels.
**Histogram of Oriented Gradients (HOG):** The purpose of HOG is to detect the presence of a particular object oriented at a specified direction [36]. The magnitude of pixel orientation data is weighted to establish the criteria for characterizing an item in these attributes.
**Gabor Filters:** They are linear Gabor filters that detect if an image has a certain frequency content within a given region of interest for texture research [37]. Many current vision experts believe that the frequency and orientation representations of Gabor filters are like those perceived in the human eye.
**Gray Level Cooccurrence Matrix (GLCM):** A GLCM is a matrix representing the frequency of cooccurrence of a pair of pixel intensities at a specified distance and angle [38]. The GLCM is computed to extract the texture features from images. Cluster prominence, cluster shade, dissimilarity, energy, entropy, homogeneity, and maximum probability are the GLCM features used in this study.

### 4.3. Deep-Learning Approaches

Currently, deep-learning approaches, particularly convolutional neural networks, have been extensively employed in computer-aided medical diagnostics. In this study, two deep-learning models are employed via transfer learning including ResNet50 and DenseNet. Moreover, a custom-developed CNN model is proposed.

**Figure 4:**
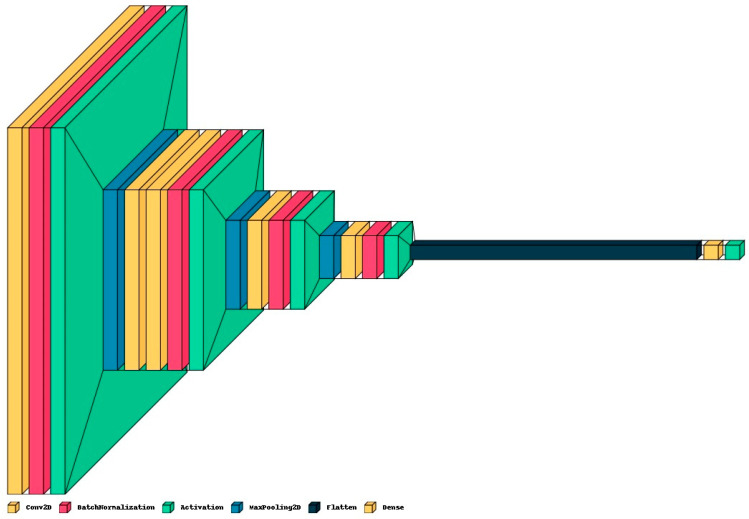
The architecture diagram of proposed model for DFU classification.

**Figure 5:**
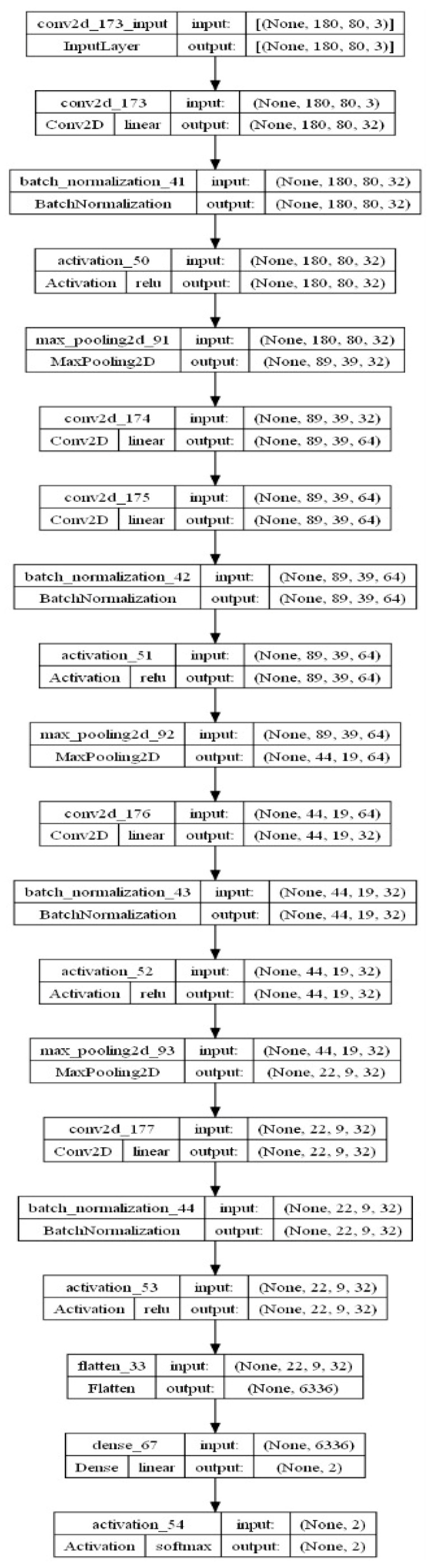
The complete architecture working map of the proposed model for DFU classification.

**ResNet50:** This model [39] was originally trained for 1000 classes using the ImageNet database [40]. The ResNet50 CNN model is adequately efficient on vision tasks, and particularly fits well considering the efficiency as well as complexity. Moreover, this has been widely used as the default choice for deep transfer learning in computer vision tasks. It has 48 convolutional layers, one max-pool, and one average-pool layer. For diabetic foot ulcer recognition, the last three layers’ parameters were updated via transfer learning by network training using thermogram image data. The input image was resized as 224 × 224 to match the ResNet input image resolution requirement. The output was restricted to one neuron, providing the probability for the sample to be recognized as a diabetic group.
**DenseNet121:** DenseNet was developed with the aim to obtain benefit from a deep network while keeping fewer parameters [41]. It improves the accuracy by minimizing the problem of a vanishing gradient. It has 120 convolutional layers and 4 average-pool layers. To use it with diabetic foot thermogram data, the last two layers were used for the parameter update via training. The input image was resized at 224 × 224 for this network also.
**Proposed CNN Model:** In addition to pre-trained networks, a custom CNN model was developed specifically for DF classification. For this purpose, the DFTNet model was adopted as the base model [9]; however, there were major differences. This study utilized the input volume size 180 × 80 × 3; however, the DFTNet used an input volume of 227 × 227 × 3. The learning rate was chosen as 0.001 with the Adam optimizer. The batch size was set to 64. The network architecture diagram is shown in Figure 4. The detailed working of the proposed model is shown in Figure 5.

## 5. Results and Discussion

For training the classifiers, 80% of the data were used for training and the remaining 20% for test purposes. For performance evaluation, classification measures such as the sensitivity, specificity, precision, accuracy, and F1-score were considered. The mathematical expressions of these measures are as follows:

Sensitivity = TP/(TP + FN)(1)

Specificity = TN/(TN + FP)(2)

Accuracy = (TP + TN)/(TP + TN + FP + FN)(3)

F1 Score = 2TP/(2TP + FP + FN)(4)

where TP: true positive, TN: true negative, FP: false positive, FN: false negative.

### 5.1. Results of Machine-Learning Approaches

As discussed in the methodology section, the machine-learning approaches used for the classification of thermogram data include SVM, RF, XGBoost, naive Bayes, ADABoost, kNN, and bagging. To compute the results for each of these algorithms, all individual features including HOG, Gabor, GLCM, and LBP were employed. Moreover, a combination of all features was also used.

#### 5.1.1. Classification of Combined Data (Image+Patch)

**Figure 6:**
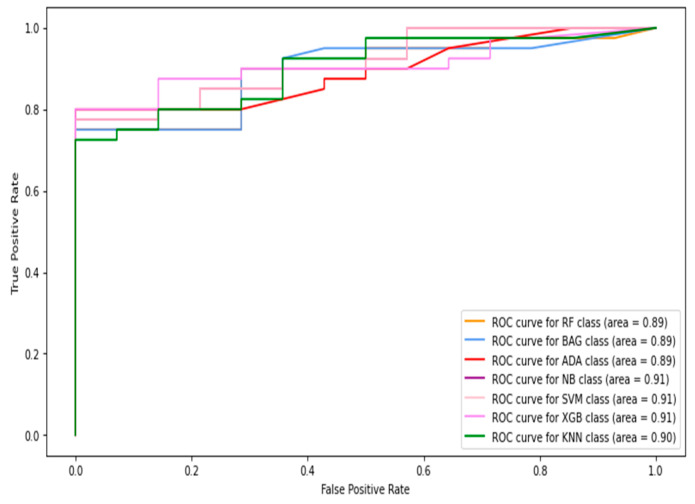
The ROC curves of classifiers using HOG feature (image + patch).

**Figure 7:**
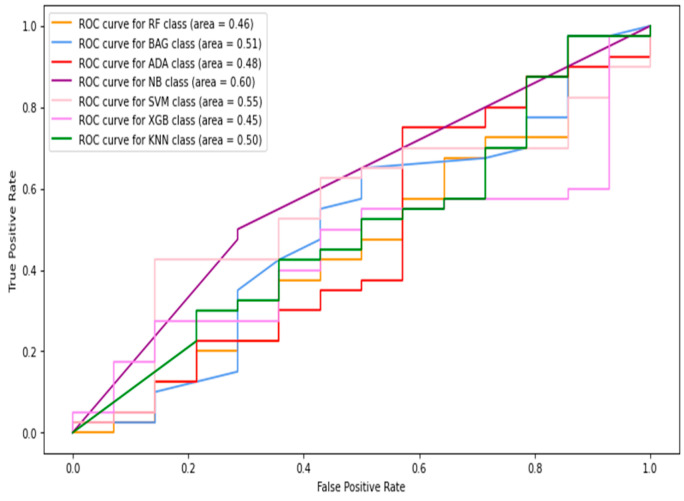
The ROC curves of classifiers using Gabor feature (image + patch).

**Figure 8:**
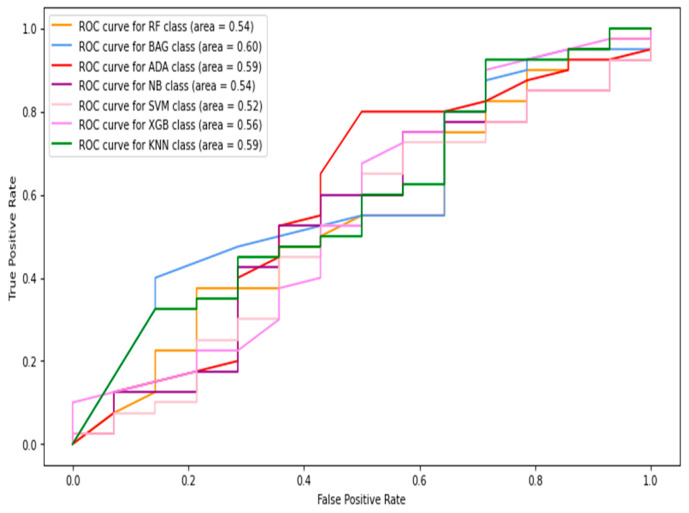
The ROC curves of classifiers using GLCM feature (image + patch).

**Figure 9:**
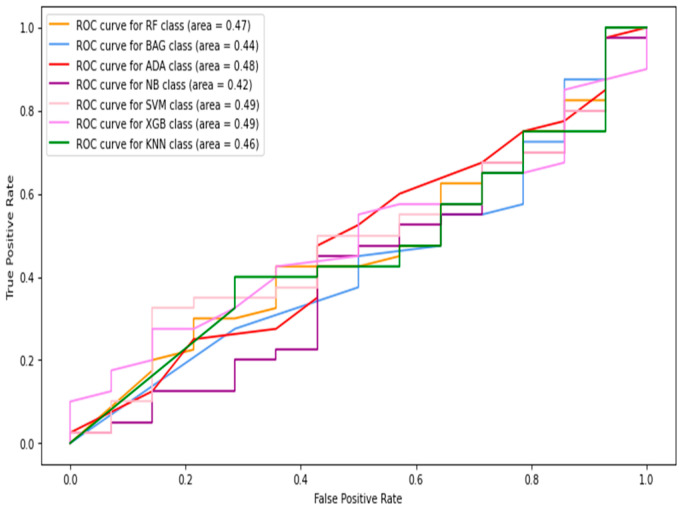
The ROC curves of classifiers using LBP feature (image + patch).

**Figure 10:**
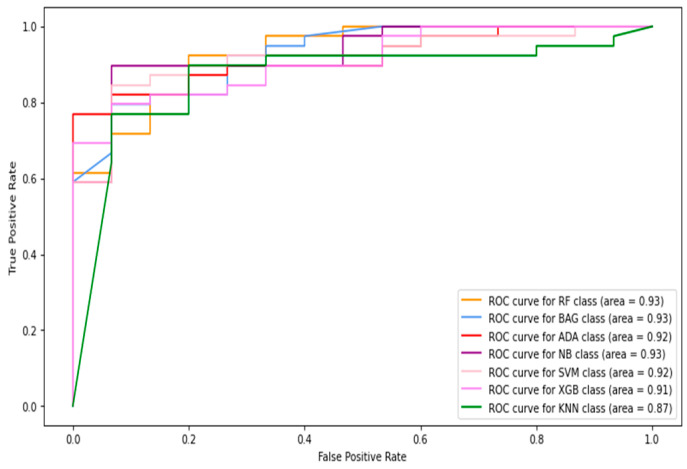
The ROC curves of classifiers using HOG + Gabor + GLCM + LBP feature (image + patch).

In this section, the images and patches are combined to form the total data. Then, the features are extracted from these data and used for classification. The receiver operative characteristic (ROC) curve and AUC (area under the curve) results of the machine-learning classifiers are shown in Figure 6, Figure 7, Figure 8, Figure 9 and Figure 10, using individual as well as combined features. Table 2 shows the results in terms of the evaluation metrics for the individual and combined features. It can be observed that a sensitivity score of 0.71 was recorded using the SVM, RF, XGBoost, and bagging classifiers with HOG features. The SVM and bagging produced the best results using both the HOG features and Gabor features. In the case of specificity, again, the SVM produced 0.95 using the GLCM features. The overall best accuracy of 78% was achieved by the SVM classifier while combining all four kinds of features. The highest F1-score was recorded by both the RF and XGBoost classifiers of 0.71. By comparing the ROC curves of classifiers employing different features, it can be observed that the HOG features produced the best results among the other individual features. The overall best AUC of 0.93 was recorded by employing combined features from multiple classifiers including RF, bagging, and naive Bayes. Concretely, the SVM happened to be the best machine-learning classifier regarding recognizing the diabetes class (with the highest sensitivity value observation), while the combined features gave the best accuracy as well as AUC.

#### 5.1.2. Full-Image Thermogram-Based Classification Results

**Figure 11:**
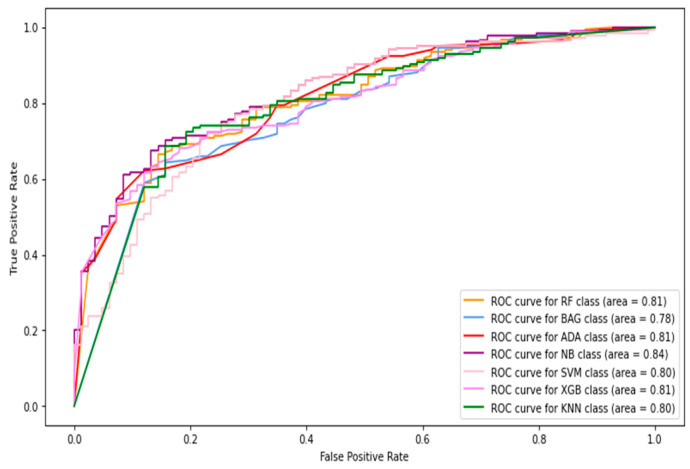
The ROC curves of classifiers using HOG + Gabor + GLCM + LBP feature (full images only).

In this section, thermogram image-level data are considered to record the classification results. Considering the results presented in the previous section, further experiments with machine-learning classifiers were restricted to using combined features only. Therefore, the HOG, Gabor, GLCM, and LBP features were extracted from the images and combined to be fed into the classifiers. Table 3 shows the image-level classification results using the combined features. The ROC curves are shown in Figure 11. The best sensitivity 0.642 was recorded using the kNN classifier while the SVM produced the best specificity value of 0.968. The XGBoost classifier achieved the best results for image-level classification with an 85.6% accuracy and F1-score of 0.688. The highest AUC of 0.84 was recorded with the naive Bayes classifier.

#### 5.1.3. Thermogram Patch-Based Classification Results

**Figure 12:**
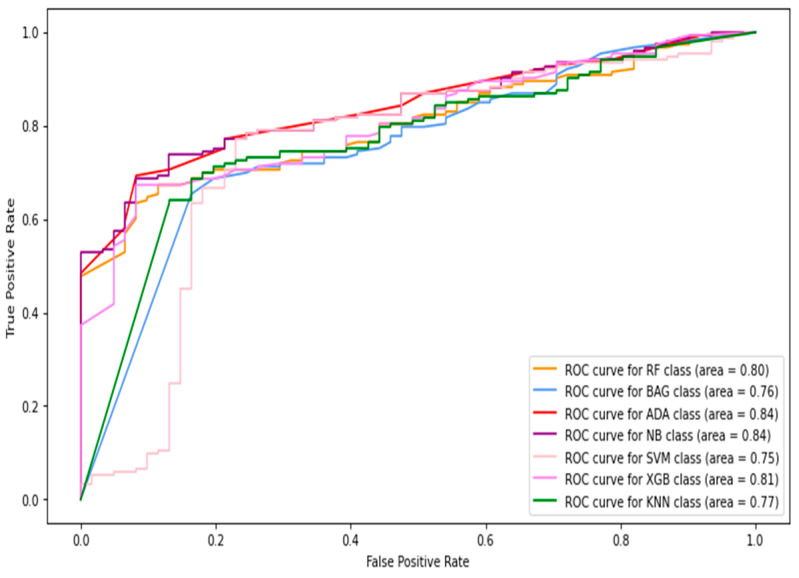
The ROC curves of classifiers using HOG + Gabor + GLCM + LBP feature (patches only).

In this section, only thermogram patch-level data are considered to compute the results. The HOG, Gabor, GLCM, and LBP features were extracted from the image patches and combined to be fed into the classifiers. Table 4 shows the image-level classification results using the combined features. The AdaBoost classifier produced the best sensitivity score and F1-score of 0.638 and 0.609. In contrast, the naive Bayes classifier achieved the highest specificity and accuracy of 0.935 and 78.7%, respectively. The ROC curves of the patch-level results are shown in Figure 12. The highest AUC of 0.84 was observed by the naive Bayes classifier.

### 5.2. Results of Deep-Learning Approaches

**Figure 13:**
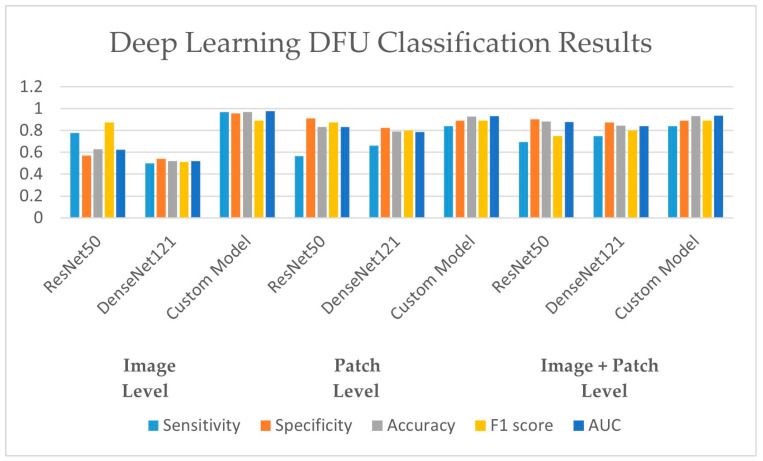
DFU classification results using deep-learning approaches.

**Figure 14:**
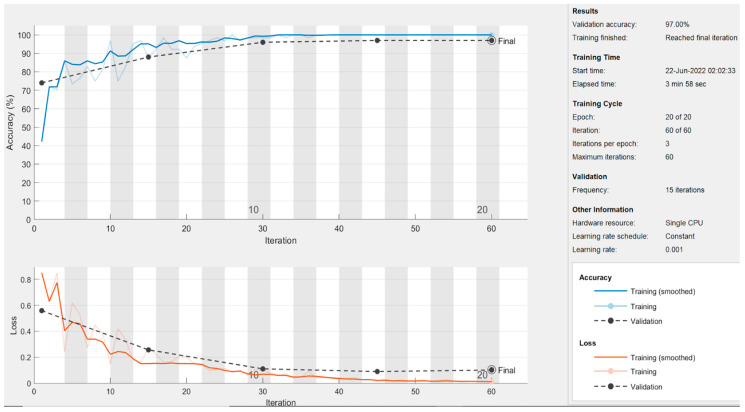
Full-image-level results of the proposed model.

**Figure 15:**
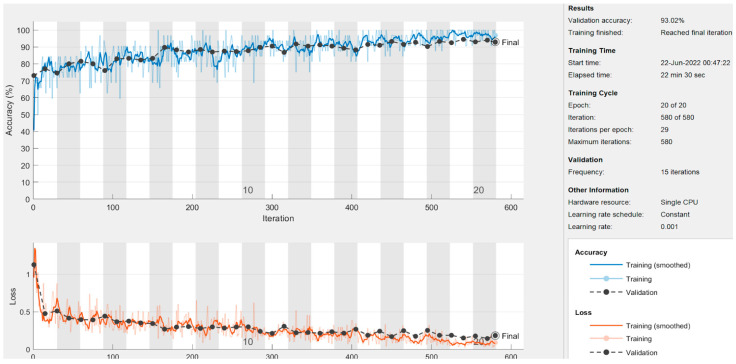
Patch-level results of the proposed model.

**Figure 16:**
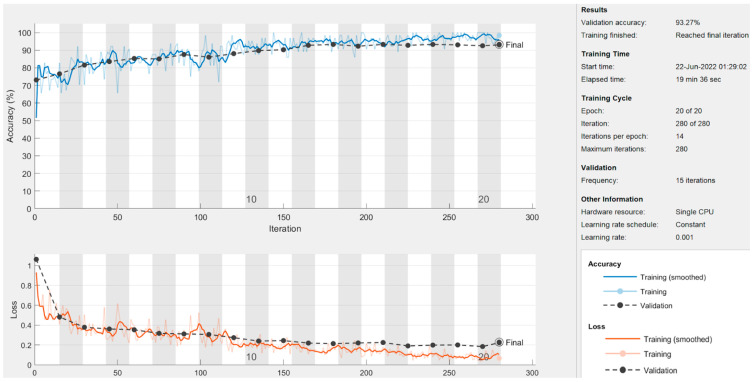
Image–patch level results of the proposed model.

In this section, the results of the CNN-based models are presented. As discussed earlier in the methodology section, the ResNet50 and DesnseNet121 models are used via transfer learning to classify the thermogram data. Moreover, the customized CNN model was developed for DFU classification. For the deep-learning approaches, the results are recorded at image level, at patch level, and by combining the images and patches. The results of both pre-trained models as well as the customized model are shown in Table 5. It can be observed that the proposed CNN model outperformed the pre-trained models in terms of sensitivity at each data representation level while achieving a best value of 0.97 at image level. The ResNet50 model produced the best specificity at patch level and at image–patch level of 0.913 and 0.902, respectively. However, the highest specificity of 0.958 was achieved by the proposed model again. The proposed model achieved the best accuracy among the three deep-learning models with a value of 97.1%, 93%, and 93.3% at image level, patch level, and image-patch level, respectively. The best F1-score of 0.891 was again recorded for the proposed model for all data representation levels. In medical diagnosis, it is critical to reduce false negatives so that the potential patient may be treated on time and the risk of amputation may be reduced. Table 5 presents the sensitivity and specificity analysis of the pre-trained models as well as the custom model. It can be observed that the custom model achieved the highest sensitivity rate and ensured the minimization of false negatives at all three levels. The graphical representation of the results using deep-learning-based models are shown in Figure 13. The accuracy and loss observations for the proposed CNN model are illustrated in Figure 14, Figure 15 and Figure 16 at image level, patch level, and image-patch level, respectively. The proposed custom-developed CNN model was trained on the original foot thermogram data from scratch. In comparison, the pre-trained model was utilized with pre-trained weights originally tuned on ImageNet data; only the weights of the last couple of layers were tuned based on the thermogram data. Therefore, the accuracy of the custom-developed model was better than the pre-trained models.

When comparing the results of the proposed model with the state-of-the-art existing solution [9], the comparison is not straight forward. Firstly, the number of samples used after augmentation with the DFTNet were 10× than the original samples; however, the samples after augmentation were almost 3× which means that only 1/3 of data were used in this study in comparison to [9]. Next, only the patch-level thermogram data were considered in the study [9]. However, this study used data representation and result computation at image level, patch level, and image-patch level. Moreover, this study presents a binary classification between the diabetic foot and control group foot. However, the study [9] segregated the data as five distinct classes, and performed binary classification by taking two classes at a time randomly. Yet, they [9] achieved the best AUC score of 0.8533 and 0.8333 using the DFTNet and ANN, respectively. In comparison, we achieved an AUC score of 0.976, 0.932, and 0.938 for the image-level, patch-level, and image-patch level data, respectively. The DFTNet [9] achieved the best accuracy of 0.853 while the proposed model produced a 0.97 accuracy. Concretely, the comparison with the study [9] is not simple and there are many differences with respect to data selection, data quantity, data representation, and class representation. However, the proposed model outperforms by looking at several possible simpler comparisons.

Regarding the limitations of this study, it is worth mentioning that in all the experiments, including machine-learning and deep-learning classification, the thermogram images of the database and the augmented samples were utilized without any pre-processing. However, since the augmentation was performed only by rotation and flipping, the content of the image was retained. Speaking about the practical applicability of the proposed model, it cannot replace a human expert completely; however, it can provide a reliable second opinion while diagnosing the diabetic foot ulcer diagnosis. In contrast, in remote areas where a medical expert is not available, such a system can play a significant role in decision-making for DFU diagnosis.

## 6. Conclusions

In this study, the classification of DFU foot is presented via the comprehensive and comparative analysis of machine-learning as well as deep-learning approaches. The thermogram data of DFU and non-DFU feet are used at image level, patch level, and combined image–patch levels. The results of the machine-learning approaches are presented by employing several features extracted from thermograms. The SVM classifier among the machine-learning techniques proved to be the best. The classification results of the machine-leaning models were best when the data at the full-image level were used. Among the pre-trained and the proposed deep-leaning models, the proposed model produced the best results. The comparison of the proposed model with an existing solution based on the used thermogram data was not straight forward. However, while comparing at an abstract level, the proposed model performed better given the constraints.

## Figures and Tables

**Table 1:** Thermogram image data detail: original and after augmentation.

Category	Diabetic Group	Control Group	Total
No. of cases	122	45	167
Original full images	244	90	334
Images after augmentation	500	500	1000
Original patches	976	360	1336
Patches after augmentation	1500	1500	3000

**Table 2:** Machine-learning classifier results using different features for combined image+patch data.

Evaluation Metric	Features	SVM	RF	XGBoost	Naive Bayes	AdaBoost	kNN	Bagging
**Sensitivity**	**HOG**	**0.71**	**0.71**	**0.71**	0.64	0.57	0.67	**0.71**
	**Gabor**	**0.71**	0.14	0.16	**0.71**	0.28	0.14	**0.71**
	**GLCM**	0.26	0.28	0.36	0.14	0.14	0.36	0.4
	**LBP**	0.07	0.14	0.14	0.07	0.07	0.14	0.19
	**Combined**	0.53	0.51	0.51	0.46	0.64	0.57	0.55
**Specificity**	**HoG**	0.85	0.9	0.9	0.85	0.87	0.7	0.87
	**Gabor**	0.9	0.77	0.87	0.47	0.8	0.9	0.87
	**GLCM**	**0.95**	0.75	0.75	0.85	0.9	0.75	0.77
	**LBP**	0.9	0.78	0.78	0.8	0.85	0.73	0.88
	**Combined**	0.89	0.83	0.82	0.94	0.79	0.81	0.83
**Accuracy**	**HoG**	0.71	0.65	0.69	0.66	0.59	0.7	0.73
	**Gabor**	0.68	0.61	0.65	0.54	0.67	0.7	0.73
	**GLCM**	0.7	0.63	0.65	0.67	0.6	0.65	0.67
	**LBP**	0.69	0.59	0.6	0.61	0.65	0.57	0.59
	**Combined**	**0.78**	0.73	0.72	0.79	0.75	0.73	0.74
**F1-score**	**HoG**	0.67	**0.71**	**0.71**	0.62	0.59	0.6	0.69
	**Gabor**	0.7	0.16	0.1	0.44	0.3	0.2	0.69
	**GLCM**	0.34	0.29	0.34	0.18	0.2	0.34	0.36
	**LBP**	0.1	0.16	0.19	0.08	0.09	0.15	0.17
	**Combined**	0.59	0.54	0.53	0.57	0.61	0.57	0.57

**Table 3:** Machine-learning classifier results using different features for full-image-level data.

Models	Sensitivity	Specificity	Accuracy	F1-Score
**SVM**	0.345	**0.968**	0.811	0.479
**RF**	0.559	0.948	0.850	0.652
**XGB**	0.630	0.932	**0.856**	**0.688**
**NB**	0.392	0.9	0.772	0.464
**ADA**	0.630	0.896	0.829	0.650
**kNN**	**0.642**	0.916	0.847	0.679
**BAG**	0.619	0.908	0.835	0.654

**Table 4:** Machine-learning classifier results using different features for patch-level data.

Models	Sensitivity	Specificity	Accuracy	F1-Score
**SVM**	0.530	0.886	0.776	0.594
**RF**	0.506	0.827	0.727	0.535
**XGB**	0.506	0.821	0.723	0.531
**NB**	0.457	**0.935**	**0.787**	0.571
**ADA**	**0.638**	0.794	0.746	**0.609**
**kNN**	0.566	0.805	0.731	0.566
**BAG**	0.554	0.827	0.742	0.571

**Table 5:** Deep-learning classifier results using data at different levels.

	Models	Sensitivity	Specificity	Accuracy	F1-Score	AUC
**Image** **Level**	**ResNet50**	0.778	0.571	0.627	0.875	0.623
	**DenseNet121**	0.5	0.542	0.521	0.511	0.52
	**Custom Model**	**0.97**	**0.958**	**0.97**	**0.891**	**0.976**
**Patch** **Level**	**ResNet50**	0.565	**0.913**	0.832	0.874	0.834
	**DenseNet121**	0.661	0.822	0.791	0.8	0.788
	**Custom Model**	**0.839**	0.889	**0.93**	**0.891**	**0.932**
**Image** **+ Patch** **Level**	**ResNet50**	0.697	**0.902**	0.881	0.75	0.879
	**DenseNet121**	0.75	0.875	0.843	0.8	0.841
	**Custom Model**	**0.839**	0.889	**0.933**	**0.891**	**0.938**

## Data Availability

The data presented in this study are openly available online: https://ieee-dataport.org/open-access/plantar-thermogram-database-study-diabetic-foot-complications, accessed on 13 February 2022.
